# Clinal distribution of human genomic diversity across the Netherlands despite archaeological evidence for genetic discontinuities in Dutch population history

**DOI:** 10.1186/2041-2223-4-9

**Published:** 2013-05-20

**Authors:** Oscar Lao, Eveline Altena, Christian Becker, Silke Brauer, Thirsa Kraaijenbrink, Mannis van Oven, Peter Nürnberg, Peter de Knijff, Manfred Kayser

**Affiliations:** 1 Department of Forensic Molecular Biology, Erasmus MC University Medical Center Rotterdam, P.O. Box 2040, Rotterdam 3000 CA, Netherlands; 2 Department of Human Genetics, Forensic Laboratory for DNA Research, Leiden University Medical Center, P.O. Box 9600, Leiden 2300 RC, Netherlands; 3 Cologne Center for Genomics, University of Cologne, Weyertal 115b, Cologne 50931, Germany; 4 Netherlands Forensic Institute, P.O. Box 24044, The Hague 2490 AA, Netherlands

**Keywords:** Population substructure, Genetic cline, Genome-wide diversity, SNP, Europe, Netherlands

## Abstract

**Background:**

The presence of a southeast to northwest gradient across Europe in human genetic diversity is a well-established observation and has recently been confirmed by genome-wide single nucleotide polymorphism (SNP) data. This pattern is traditionally explained by major prehistoric human migration events in Palaeolithic and Neolithic times. Here, we investigate whether (similar) spatial patterns in human genomic diversity also occur on a micro-geographic scale within Europe, such as in the Netherlands, and if so, whether these patterns could also be explained by more recent demographic events, such as those that occurred in Dutch population history.

**Methods:**

We newly collected data on a total of 999 Dutch individuals sampled at 54 sites across the country at 443,816 autosomal SNPs using the Genome-Wide Human SNP Array 5.0 (Affymetrix). We studied the individual genetic relationships by means of classical multidimensional scaling (MDS) using different genetic distance matrices, spatial ancestry analysis (SPA), and ADMIXTURE software. We further performed dedicated analyses to search for spatial patterns in the genomic variation and conducted simulations (SPLATCHE2) to provide a historical interpretation of the observed spatial patterns.

**Results:**

We detected a subtle but clearly noticeable genomic population substructure in the Dutch population, allowing differentiation of a north-eastern, central-western, central-northern and a southern group. Furthermore, we observed a statistically significant southeast to northwest cline in the distribution of genomic diversity across the Netherlands, similar to earlier findings from across Europe. Simulation analyses indicate that this genomic gradient could similarly be caused by ancient as well as by the more recent events in Dutch history.

**Conclusions:**

Considering the strong archaeological evidence for genetic discontinuity in the Netherlands, we interpret the observed clinal pattern of genomic diversity as being caused by recent rather than ancient events in Dutch population history. We therefore suggest that future human population genetic studies pay more attention to recent demographic history in interpreting genetic clines. Furthermore, our study demonstrates that genetic population substructure is detectable on a small geographic scale in Europe despite recent demographic events, a finding we consider potentially relevant for future epidemiological and forensic studies.

## Background

The presence of genetic gradients across Europe has been described and discussed for more than 30 years. In the case of autosomal markers, a southeast to northwest gradual change in the distribution of the genetic diversity has been reported using principal component analysis (PCA) [1,2]. Initially, this gradient was described from classical markers such as blood groups [1], and later was confirmed by genome-wide single nucleotide polymorphisms (SNPs) [3,4]. This genetic diversity cline is traditionally explained by several major prehistoric demographic events in Europe: the first colonization of Europe by anatomically modern humans together with a postglacial re-expansion from the southern European refugee areas in Palaeolithic times, and the introduction of the Neolithic agricultural lifestyle by people from the Near East [1]. Theoretical studies using computer simulations [5] have shown that such major prehistoric demographic events can produce genetic gradients in autosomal markers that in particular situations resemble what is observed in real data from Europe. However, simulations tend to necessarily simplify the demographic history by ignoring more subtle demographic events that took place throughout history at a smaller geographical scale such as those in Europe [6]. Furthermore, it was suggested that caution should be taken when interpreting results from PCA analyses [7]. With this study we aim to investigate whether (similar) spatial patterns in genomic diversity can also be detected on a micro-geographic scale, within a European country like the Netherlands, and if so, whether these patterns could also be explained by more recent demographic events.

**Figure 1:**
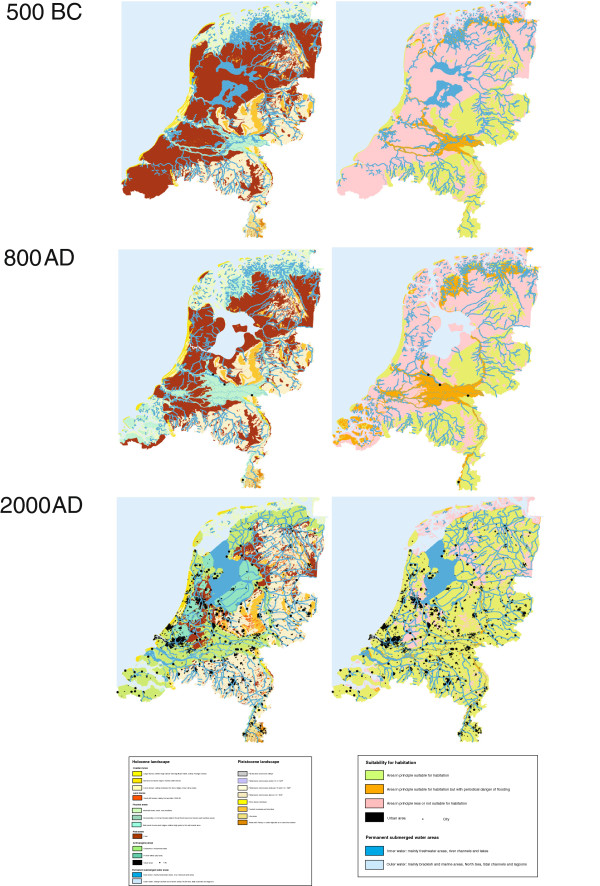
**Paleogeographic maps of the Netherlands.** The region comprising the Netherlands depicted via paleogeographic maps indicating the different natural landscapes (left panels) occurring in 500 BC, 800 AD and 2000 AD, and inferred suitability for human habitation (right panels) at the same time periods. For further explanation including color code see inbuilt legend.

We chose the Dutch population as an example because results from geological, archaeological, and historical studies strongly indicate that during several prolonged periods of time different factors and events resulted in discontinuities of human populations on the current territory of the Netherlands. A summary of the population history of the Netherlands is provided in the supplementary material [see Additional file 1]. In brief, geological processes have been a major driving force shaping the Dutch demographic history. During prehistory, the Dutch landscape went through several significant transformations. Also in more recent times, mainly under influence of variable water levels of the North Sea and many rivers, the Dutch landscape underwent major changes. Humans additionally had a substantial direct impact on the Dutch landscape with major land-reclamation projects [8]. As a result of this, large parts of the country that are densely populated today, were not suitable for human habitation during several periods in both prehistoric and historic times [9,10]. Figure 1 provides examples of the changing Dutch landscape and its suitability for human settlement from 500 Before Christ (BC) to the present day, and illustrates the changing conditions relevant for human habitation. Furthermore, archaeological and historical evidence provides several indications for cultural processes that additionally caused discontinuity of the Dutch population. Some examples are i) the fast population growth and subsequent decline during the Roman period (from 15-30,000 to 150,000 to less than 40,000 in just 400 years time) followed by substantial subsequent migrations [10-15], ii) the religious division that emerged in 1648, which has lasted to the present day [15], and iii) the substantial and complex migrations during the second half of the 20th century [16] [see Additional file 1 for more details].

Given the archaeological, geological and historical evidence for genetic discontinuity in Dutch population history, one might expect that the ancient genetic signatures from Palaeolithic and Neolithic times, such as the southeast to northwest cline seen across Europe, would not be detectable in the contemporary Dutch gene pool. To test this hypothesis via studying the spatial distribution of the Dutch genomic diversity, including computer simulations, and to investigate the overall genomic-geographic substructure of the Dutch population, we sampled 999 individuals at 54 sites across the Netherlands following a grid-like scheme. DNA samples were genotyped with the Genome-Wide Human SNP Array 5.0 (Affymetrix; *http://www.affymetrix.com/estore/*) from which 443,816 quality-controlled genome-wide autosomal SNPs were used in various spatial, cluster, and simulation analyses.

## Methods

### Samples

**Figure 2:**
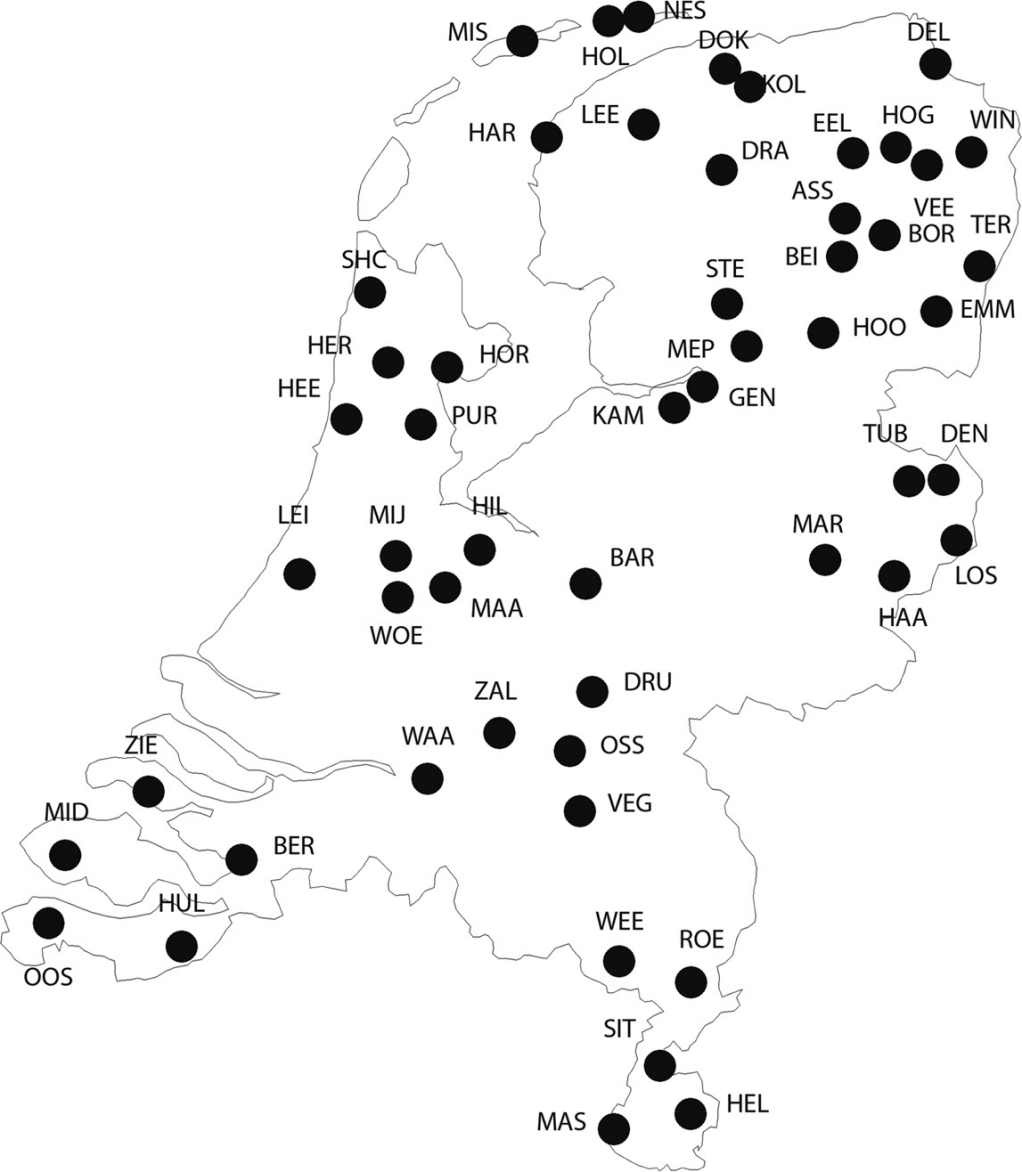
**Sampling locations within the Netherlands.** Map of the 54 geographic sites the 999 Dutch individuals were collected from across the Netherlands under a grid-like sampling scheme.

A total of 999 male blood donors with self-defined Dutch ancestry sampled from 54 geographic regions across the Netherlands (Figure 2) by mostly excluding major cities to avoid very recent admixture effects (see Table 1) were purchased from Sanquin, the only official Dutch blood-collecting organization. All samples come from healthy blood-donor volunteers who regularly (once or twice per year) donate blood. Sanquin is exclusively authorized by the Dutch government to sell and or distribute products derived from these donated blood samples. For the purpose of this study all donors were asked, prior to their donation, if they agreed with the sales of part of their white cells to the Forensic Laboratory for DNA Research (FLDO) of the Leiden University Medical Center for fundamental population genetic research purposes. They were given sufficient time to read an informed consent and explanation document prior to their donation. Consent was registered by Sanquin, and only the samples from donors who agreed were subsequently sold to the FLDO. Genetic projects of this kind (strictly anonymous and commercially purchased from a third party) fall outside the evaluation scope of the LUMC ethics committee, hence this project was not formally evaluated. Prior to the study, the FLDO and Sanquin discussed the possibility to only receive samples from known unrelated donors residing in specific Dutch towns and cities. This enabled a more-or-less even coverage of the total Dutch area. Samples were received anonymously, with only the place of residency of the donor indicated. Participation was restricted to males. As such, 2,100 Dutch male samples were collected. The 999 males studied and described here represent a geographically random subset of this total set of 2,100 males collected.

**Table 1:** **Dutch subpopulations studied, their sampling coordinates, and sample size before and after data cleaning**^**a**^

**Sampling site**	**Code**	**Latitude**	**Longitude**	**N initial**	**N clean**
Assen	ASS	53	6.55	17	17
Barneveld	BAR	52.1333	5.58333	20	20
Beilen	BEI	52.8667	6.51667	9	9
Bergen op Zoom	BER	51.5	4.3	18	18
Borger	BOR	52.9167	6.8	7	7
Delfzijl	DEL	53.3333	6.91667	10	10
Denekamp	DEN	52.3833	7	22	19
Dokkum	DOK	53.3333	6	23	23
Drachten	DRA	53.1	6.1	20	20
Druten	DRU	51.8833	5.61667	20	18
Eelde	EEL	53.117	6.583	7	7
Emmen	EMM	52.7833	6.9	9	9
Genemuiden	GEN	52.6333	6.05	16	16
Haaksbergen	HAA	52.15	6.73333	22	21
Harlingen	HAR	53.1833	5.41667	23	20
Heemskerk	HEE	52.5167	4.66667	18	18
Heerhugowaard	HER	52.6667	4.85	21	21
Heerlen	HEL	50.9	5.98333	20	20
Hilversum	HIL	52.2333	5.18333	20	20
Hollum	HOL	53.4167	5.63333	10	8
Hoogeveen	HOO	52.7333	6.48333	24	21
Hoogezand	HOG	53.1667	6.76667	1	1
Hoorn	HOR	52.65	5.06667	20	20
Hulst	HUL	51.283	4.05	21	20
Kampen	KAM	52.55	5.91667	24	24
Kollum	KOL	53.2833	6.15	11	11
Leeuwarden	LEE	53.2	5.78333	23	23
Leiden	LEI	52.15	4.5	68	65
Losser	LOS	52.2667	7.01667	20	20
Maarssenbroek	MAA	52.133	5.033	20	20
Maastricht	MAS	50.85	5.68333	20	19
Markelo	MAR	52.2333	6.5	17	16
Meppel	MEP	52.7	6.2	24	24
Middelburg	MID	51.5	3.617	19	19
Midsland	MIS	53.3833	5.28333	20	20
Mijdrecht	MIJ	52.2	4.86667	20	19
Nes	NES	53.45	5.76667	6	5
Oostburg	OOS	51.333	3.5	18	18
Oss	OSS	51.767	5.534	20	18
Purmerend	PUR	52.5167	4.95	20	19
Roermond	ROE	51.2	6	21	21
Schagen	SCH	52.7833	4.8	20	19
Sittard	SIT	51	5.867	20	20
Steenwijk	STE	52.7833	6.11667	11	11
Ter Apel	TER	52.8833	7.06667	6	6
Tubbergen	TUB	52.4167	6.78333	18	16
Veendam	VEE	53.1	6.88333	19	19
Veghel	VEG	51.6167	5.55	20	20
Waalwijk	WAA	51.6833	5.06667	20	20
Weert	WEE	51.25	5.71667	20	20
Winschoten	WIN	53.15	7.03333	15	15
Woerden	WOE	52.0833	4.91667	21	21
Zaltbommel	ZAL	51.8	5.25	20	20
Zierikzee	ZIE	51.65	3.916	20	18

^a^See Methods for details on data cleaning.

### Genome-wide data

Each individual was genotyped with the GeneChip Human Mapping 500 K Array Set (Affymetrix) and genotypes were inferred with the Bayesian Robust Linear Model with Mahalanobis distance classifier (BRLMM) algorithm. Sampling sites were not considered in the microarray genotyping procedure to avoid batch effects. Individual data cleaning was performed using the Tukey’s approach as applied in Lao *et al*. [3]. In brief, for each pair of individuals, an identical-by-state (IBS) distance was computed. Within each subpopulation, Tukey’s outlier criterion was applied and individuals either showing large distances to the rest of the individuals of the same subpopulation (genetic outliers), or individuals of pairs showing smaller distances than the observed in all the pairs of the same subpopulation (strongly genetically related), were excluded. It must be noticed that, due to limited sample size in each subpopulation, the power to detect individual genetic outliers can be small. Using this approach, 30 individuals did not pass the quality control and were excluded. SNPs with more than 10% of missing genotypes in at least one subpopulation were also excluded. Hardy-Weinberg Equilibrium (HWE) was tested in all the autosomal SNPs for each subpopulation. SNPs that did not pass HWE in at least one subpopulation after multiple testing were excluded. Of the 443,816 markers, 414,633 autosomal SNPs were considered clean after applying this filter. None of the considered individuals showed a percentage of missing genotypes >2% and therefore there was no further individual exclusion. We next pruned for linkage disequilibrium (LD) by means of ascertaining markers that showed low LD at a distance <500 kb. We computed Kendall’s Tau B statistic [17] using the contingency table computed between the genotypes of two loci at a distance <500 kb. We included new loci in the final dataset if the absolute value of the statistic was smaller than 0.5 with the ones already included. After LD ascertainment, the number of autosomal markers was 137,662. This set of markers and 969 individuals were used in further analyses, except in the case of the spatial ancestry analysis (SPA) [18]), where 952 individuals and all the (non-LD pruned) SNPs were used. Data are available for nonprofit research via an institutional website [19].

### Data analyses

An IBS distance matrix between pairs of individuals was computed and plotted by means of classical multidimensional scaling (MDS) as implemented in the cmdscale routine of R software [20]. Identical-by-descendent (IBD) genomic regions between pairs of individuals were estimated with the fastIBD algorithm [21] as implemented in BEAGLE [22] using default settings. A normalized IBD shared length between pairs of individuals was then computed using the approach proposed by Gusev *et al*. [23]:

$$W_{\mathrm{ij}}=\frac{1}{W_{\mathrm{tot}}}\sum_{r\in K}\sum_{t=K_{\mathrm{pi}}^{r}}^{K_{\mathrm{pe}}^{r}-1}F\left(t\right)$$

where W_ij_ is a value ranging from 0 (that is, no sharing) to 1 (that is, sharing of the whole genome) between individuals *i* and *j*. W_tot_ is the maximum value of sharing that can be obtained:

$$W_{\mathrm{tot}}=\sum_{s=1}^{n}F\left(s\right)$$

and *F(t)* is the normalized length of an interval between two SNPs, and it weighs the length of the fragment by the number of individuals sharing the segment:

$$F\left(s\right)=\left\{\begin{array}{l} \frac{l\left(s,s+1\right)}{\pi \left(s,s+1\right)}\mathrm{if}\;\pi \left(s,s+1\right)\neq 0,\\ 0\;\text{otherwise} \end{array}\right)$$

A distance measure was obtained by setting 1-W_ij_ for all the pairs. The distance matrix was plotted by means of MDS after adding a constant to the matrix in order to make all the eigenvalues positive [24]. The mean of the first two dimensions by population were compared with the geographic coordinates of the sampling sites by means of a procrustes analysis [25] as implemented in the protest method of the vegan R package.

Proportions of ancestry for each individual were computed using ADMIXTURE [26] and FRAPPE [27], setting the number of groups (*K*) to 1 to 6. A pie chart map was constructed for *K* = 5 on ADMIXTURE consensus results (out of 10 independent replicates merged with CLUMPP [28] using the greedy algorithm implemented in the software) using MapViewer software [29]. CLUMPP [28] was used to perform a comparison of the outcome of the two clustering algorithms.

A spatial autocorrelogram was computed using the method proposed by [30]. First, a *D*^2^ distance and covariance matrix between pairs of individuals is computed. *d*_*ij*_^2^ between individual *i* and individual *j* is defined as:

$$d_{\mathrm{ij}}^{2}=\frac{\sum_{s=1}^{n}{\left(G_{\mathrm{is}}-G_{\mathrm{js}}\right)}^{2}}{n}$$

where *G*_*.s*_ is the not null genotype (taking values 0 for AA,1 for AB and 2 for BB [30]) of individual at snp *s* and *n* is the total number of SNPs for which either individual *i* and individual *j* do not contain null genotypes.

The covariance *c*_*ij*_ between *i* and *j* was computed as:

$$c_{\mathrm{ij}}=\frac{\left[-d_{\mathrm{ij}}^{2}+\frac{\left(\sum_{j=1}^{N}d_{\mathrm{ij}}^{2}+\sum_{i=1}^{N}d_{\mathrm{ij}}^{2}\right)}{N}-\frac{\left(\sum_{i\neq j}^{N}d_{\mathrm{ij}}^{2}\right)}{N^{2}}\right]}{2}$$

in formula 13 of [30].

The covariance matrix was used to perform a genetic based spatial autocorrelation analysis [30]. We considered 24 distance classes. Overall significance of the autocorrelogram was tested by means of shuffling the individuals at random between the subpopulations and computing the r value for each class distance. We applied the method described by [31] to propose a combined *P* value of the autocorrelogram.

To model the genotypes of each individual in two dimensions, we performed a spatial structure analysis (SPA) [18] with SPA software (http://genetics.cs.ucla.edu/spa/). This method attempts to model the allele frequency of each marker as a function of geographic positioning, and then places the individuals in this defined space. SPA was conducted on all SNPs in order to identify genomic regions showing steep allele frequency gradients. Genomic regions showing an excess of large scores for selection signal detection were detected by means of local Moran’s I statistic [32]. Local Moran’s I statistic was computed taking a window size of 50 kb at each side of the considered marker:

$$I\left(s_{i}\right)=\frac{n}{n\left(n-1\right)S^{2}}\left(Z_{i}-\bar{Z}\right)\sum_{j=1}^{n}w_{\mathrm{ij}}\left(Z_{j}-\bar{Z}\right)$$

where *i* is the marker of interest, *n* is the number of markers that are within a distance <50 kb of the marker of interest, Z_*i*_ is the computed SPA value of the marker *i* and *w*_*ij*_ is the weight between marker *i* and *j* (1 if the marker is within the window of 50 kb, otherwise 0). Local Moran’s I statistic takes positive values (indicating positive local autocorrelation) if the value of one SNP is extreme compared to the rest of the genome and it is surrounded by SNPs with values of similar magnitude. A *P* value was computed by reshuffling the value of the score 1,000 times at random, then computing local Moran’s I statistic for each marker and comparing it with the observed one. A Manhattan plot of the Local Moran’s I statistic value for these markers with a *P* value <5e-04 was computed using mhtplot function of the gap R package [33].

We computed Weir and Cockerham’s combined Fst [34] between pairs of subpopulations with more than 10 individuals (comprising 46 populations). Negative Fst values between pairs of subpopulations were set to 0. Classical multidimensional scaling was performed with this matrix after adding a constant [24] to prevent negative eigenvalues. Procrustes [25] was used to compare geographic coordinates with the first two dimensions. Dependence of the genetic distance matrix and geographic distance was assessed by means of Pearson’s correlation and the statistical significance by means of a Mantel test [35] as implemented in PASSAGE software [36] using 1,000 iterations. The presence of a geographic gradient in the Fst matrix was tested by means of a Bearing correlogram [37] using PASSAGE software [36].

### SPLATCHE2 simulations

We performed two different SPLATCHE2 [38] simulations in order to assess the impact of genetic discontinuity on the genetic landscape of the Netherlands. We used a map of Europe of 188 columns by 132 rows, sampled 39 cells from the region comprising the Netherlands and simulated 1,000 SNPs of MAF 0.03 in the Netherlands. Both simulations considered the demographic scenario described in Francois *et al*. [5] in Europe; that is, the settlement of Europe started 1600 generations ago from the Middle East by hunter-gatherers, and 400 generations ago a second expansion representing the Neolithic took place in the southeast of Europe. Carrying capacity of each cell populated by hunter-gatherers was set to 500 (50 in [5] with a cell area 9.23 times smaller) and of Neolithic by 5000 (500 in [5]). Migration rates were set to 0.4 for Palaeolithic and 0.8 for Neolithic and the growth rates to 0.5 in Palaeolithic and 0.8 in Neolithic in order to ensure the full peopling of the European continent. In the second simulation, in addition to these demographic events, we added a genetic discontinuity in the Netherlands, setting the carrying capacity of the coastal cells (representing 28 out of the 39 cells) to 0 between 70 and 35 generations ago, where they were repopulated by migrants from the neighboring populations. A distance matrix between pairs of populations based on Fst was then computed for each simulated model using Arlequin 3.1 [39], and negative Fst values set to 0. MDS analyses on each distance matrix and comparison of the MDS result with geographic coordinates of the cells was performed by means of a Procrustes analysis. A Bearing correlogram using each Fst distance matrix and geographic coordinates was conducted with PASSAGE software.

## Results and discussion

The locations of the 54 Dutch geographic subpopulations from which the 999 individuals were sampled are shown in Figure 2 and further explained in Table 1. As evident, most of the current Dutch territory was sampled evenly with an average sample size across subpopulations of 18 individuals (range between 1 and 65). Overall, about half of the genome-wide autosomal SNPs (53.75%) had a Weir and Cockerham’s Fst value [34] of zero (or smaller). The mean Fst value across all genome-wide autosomal SNPs used was only 0.003 (after setting negative values to zero) and the mean combined Fst value between pairs of subpopulations was even smaller at 0.00038. These results together demonstrate a very small overall genetic differentiation among the 54 Dutch subpopulations sampled across the entire country. In fact, genetic differentiation between geographic subpopulations from within the Netherlands as observed here is smaller than between geographic subpopulations from within other northern European countries studied thus far in a systematic fashion, such as Sweden [40]. Our genomic results are in agreement with expectations from human populations of small geographic areas, and suggest the absence of strong genetic barriers within the contemporary Dutch population (in addition to the nonexistence of strong geographic barriers). To investigate the spatial distribution of the Dutch genomic diversity as well as the genetic-geographic substructure of the Dutch population, we applied a combination of well-established and recently introduced approaches to the genomic data after stringent quality control on markers and individuals (see Methods for details on quality control).

**Figure 3:**
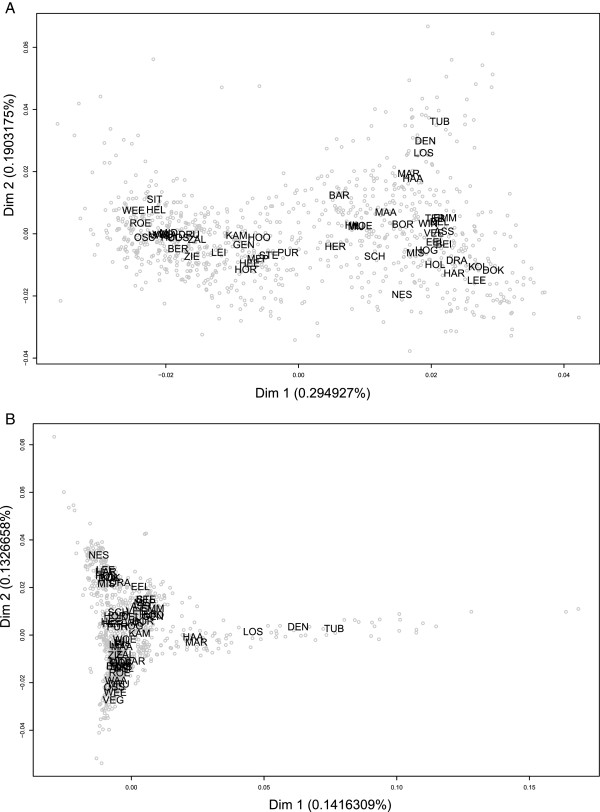
**Classical multidimensional scaling plots using identical-by-state and identical-by-descendent matrices of the Dutch samples. A)** Plot of the first two dimensions of a classical multidimensional scaling (MDS) analysis performed with the identical-by-state (IBS) distance matrix between pairs of 952 individuals using the linkage disequilibrium (LD) pruned set of genome-wide autosomal single nucleotide polymorphisms (SNPs). This set of individuals did not include 17 individuals identified by Mclust (see Methods and [see Additional file 1: Figure S2(B)]). **B)** Plot of the first two dimensions of an MDS performed using an identical-by-descendent (IBD) distance matrix between pairs of individuals. For explanation of the subpopulation abbreviations see Table 1 and Figure 2.

First, we performed a classical multidimensional scaling (MDS) analysis on an identical-by-state (IBS) distance matrix between all pairs of 969 individuals (30 individuals were excluded during quality control via the Tukey’s outlier criterion, see Method section for details). By applying Mclust [41] to the first two dimensions of this MDS [see Additional file 1: Figure S2(A) for the two-dimensional plot], we identified three clusters of individuals. The first two clusters comprised of 98.25% of all individual samples, while the third cluster comprised of 17 individuals only [see Additional file 1: Figure S2(B)]. These 17 individuals mostly represent singletons from widely dispersed geographic subpopulations: 1 from ASS, 1 from DOK, 1 from MAA, 1 from MAS, 1 from OSS, 1 from PUR, 1 from ROE, 1 from SCH, 1 from WIN, 1 from WOE, 1 from ZIE, 2 from HIL, 2 from HOO and 2 from LEI (see Table 1 for explanations of subpopulation abbreviations). Notably, these 17 individuals were mostly found contributing to differences in the first dimension of the MDS. When excluding these individuals from the MDS, the first dimension of a two-dimensional plot (accounting for 0.296% of the total variance) tends to distribute the remaining 952 Dutch individuals according to a south to north axis (Figure 3A). The mean of the first dimension in each subpopulation correlates strongly with latitude (adjusted R squared = 0.676, *P* value = 1.455e-14) and somewhat less strongly with longitude (adjusted R-squared: 0.297, *P* value = 1.214e-05). The second dimension (accounting for 0.191% of the total variance) tends to differentiate individuals from the central-east region of the Netherlands (HAA, MAR, LOS, DEN and TUB) from the rest and correlates with longitude, albeit not very strongly (adjusted R-squared = 0.297, *P* value: 1.214e-05; adjusted R-squared with latitude = 0.083, *P* value: 0.019). When considering both dimensions at the same time, the correlation in a symmetric Procrustes rotation [25] between the mean value of each dimension and the geographic coordinates of each of the 54 subpopulation was high (r = 0.762, *P* value after 1,000 simulations <0.0005), suggesting that the proposed genetic map of the Netherlands fits the geographic map of sampling locations well.

**Figure 4:**
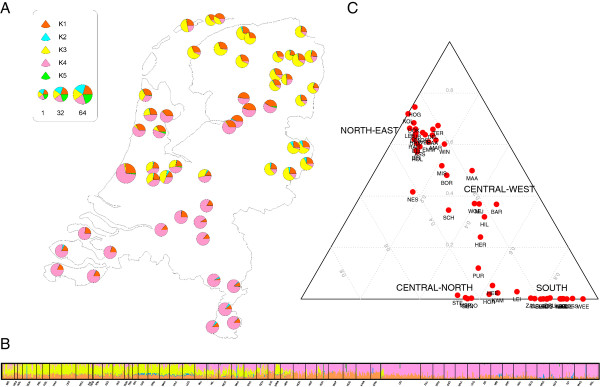
**Admixture analysis of the Dutch samples. A)** Pie chart map of the genome-wide ancestry assignment in the 54 Dutch subpopulations estimated with 10 independent runs by ADMIXTURE [26] using *K* = 5 assumed parental populations. **B)** Individual ancestry estimated by ADMIXTURE using *K = 5*. **C)** Ternary plot of subpopulations using the three most frequent (K1, K3, K4) categories identified by ADMIXTURE. For subpopulations see Table 1 and Figure 2.

Second, we performed an MDS analysis using an identical-by-descent (IBD) distance matrix between pairs of individuals (see Figure 3B for a plot of the first two dimensions). The mean of the first dimension for each subpopulation correlates with longitude (r = 0.496, *P* value: 0.0001348) while the second dimension correlates strongly with latitude (r = 0.89, *P* value: <2.2e-16). Both MDS dimensions together correlate with the geographic coordinates (Procrustes symmetric correlation: 0.679, *P* value <0.0005 based on 1,000 permutations). Furthermore, we observed a strong correlation between the MDS based on IBS and the one based on IBD (correlation in a symmetric Procrustes rotation: 0.844, *P* value = 0.001 after 1,000 permutations). Third, we carried out two genetic clustering analyses of the genomic data. In the first analysis, we used ADMIXTURE [26] allowing for *K* = 1 to 6 [see Additional file 1: Figure S3]. By performing a cross-validation error analysis [26] to distinguish the most sensible model choice, we found that at *K* = 1 the cross-validation error was smallest indicating the most sensible model and that this error increased until *K* = 4. At *K* = 5, however, the cross-validation error decayed compared to *K* = 4 and *K* = 6 [see Additional file 1: Figure S3(A)] suggesting that *K* = 5 could also be regarded as a sensible model. We therefore focused on the results of *K* = 5 in Figure 4 (the full results of *K* = 1 to 6 are available from [Additional file 1: Figure S3]). With *K* = 5, the consensus plot of ancestries (out of 10 independent ADMIXTURE replicates merged with CLUMPP [28]; H’: 1) suggests that three of the ancestral populations together describe almost all (total 97.47%: 24.53%, 30.54% and 42.4%) of the total genome-wide ancestry of the samples. The remaining two ancestral populations together represent only a very small (2.13% together, 2.07% and 0.06% separately) fraction of the average ancestry in the samples. The three main ancestry components together tend to divide the Dutch population into four main geographic groups (Figure 4C): a southern group, a northeastern group, a central-western group, and a central-northern group. In contrast, the two additional minor ancestral components appear in individuals sparsely distributed across the country (Figure 4A,B). In agreement with our MDS based on IBS and Mclust analyses, the 17 individuals classified by Mclust in the third group (and therefore removed from the subsequent MDS) tend to show a statistically significant larger ancestry component in either of these two minor ancestral populations than individuals classified into the other two groups (Wilcoxon rank sum test with continuity correction W = 815, *P* value = 2.551e-11). In the second analysis, we used FRAPPE [27], which provided a similar result to what we obtained with ADMIXTURE at *K* = 5 (H’ = 0.741 statistic from CLUMPP analysis [28]) using inferred ADMIXTURE (10 runs) and FRAPPE (one run) clustering at *K* = 5 [see Additional file 1: Figure S4 for FRAPPE results].

**Figure 5:**
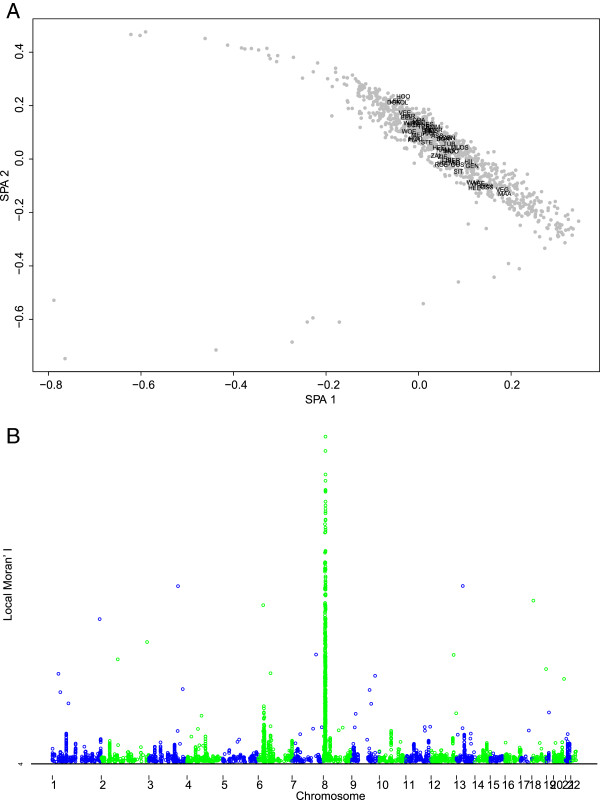
**Spatial analysis of the Dutch samples. A)** Spatial ancestry analysis (SPA). Two Dimensional Mapping of 952 Dutch individuals (gray dots) using all the single nucleotide polymorphisms (SNPs); Dutch subpopulations are placed using the mean value of the individuals for each coordinate. For subpopulations see Table 1 and Figure 2. **B)** Manhattan plot of the Local Moran’s I value computed using the steep allele frequency gradient coefficient value estimated by SPA. Only SNPs showing a statistically significant value (*P* value <0.0005) of genomic spatial association are represented.

**Figure 6:**
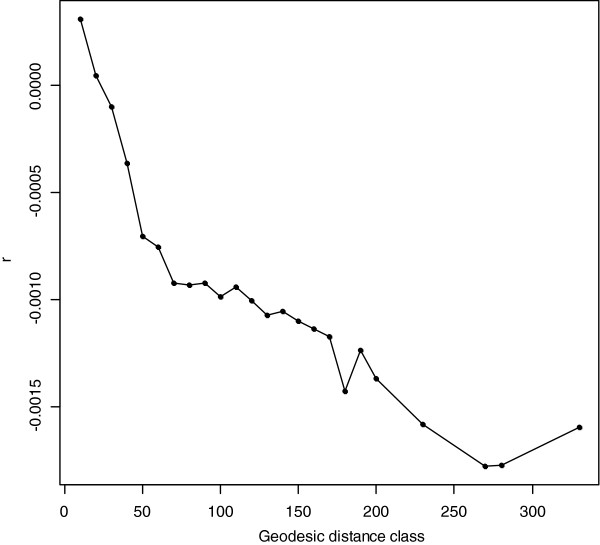
**Spatial autocorrelogram of the Dutch samples.** Spatial autocorrelogram using the pairwise covariance matrix between the 969 Dutch individuals (after data cleaning). The matrix was estimated from a modified identical-by-state (IBS) distance matrix between pairs of individuals (see Methods for details) using the subset of linkage disequilibrium (LD) pruned genome-wide single nucleotide polymorphism (SNP) markers. Geodesic distance (in km) class between individuals is plotted on the X-axis. Level of autocorrelation for each distance class is depicted on the Y-axis.

Fourth, and to further explore the geographic distribution of the genome-wide diversity across the Netherlands, we performed two different spatial analyses of the genomic data. In the first analysis, we carried out a spatial ancestry analysis (SPA) [18] on the same 952 individuals used after MDS-based outlier exclusion. As seen in Figure 5A, the Dutch individuals tend to be distributed according to a southeast to northwest gradient in a two-dimensional mapping (correlation in a symmetric Procrustes rotation between the geographic coordinates of each population and the mean value of each SPA dimension: 0.612, *P* value = 0.001 after 1,000 replications). Latitude seems to be more influenced by the second dimension of SPA (latitude = −0.578*SPA1 -7.279*SPA2 + 52.004; *P* value of SPA1 = 0.807, *P* value of SPA2 = 3.26e-06; Adjusted R-squared of the multiple linear regression: 0.655, *P* value: 6.138e-13), while both SPA dimensions seem to contribute to longitude (longitude = −11.877*SPA1 -10.616*SPA2 + 4.685; *P* value of SPA1 = 0.010025, *P* value of SPA2 = 0.000182; Adjusted R-squared of multiple regression: 0.244, *P* value: 0.0002973). We also found several regions dispersed throughout the genome with individual SNPs displaying stronger frequency gradients (*P* value <0.0005) across the Netherlands (3,627 SNPs) (Figure 5B). Of these, the most striking signal is observed at a particular region on chromosome 8 ((Figure 5B) and [see Additional file 2: Table S2]). Unfortunately, this region includes several genes so that it is difficult to conclude without additional data which of the genes may be responsible for the observed spatial pattern. Notably, we did not observe a strong SPA signal in the *LCT* gene region on chromosome 2, which is known to be involved in lactase persistency and positive selection in Europeans, or the *OCA2-HERC2* region on chromosome 15, which is known to be involved in blue-brown eye color determination and positive selection in Europeans. Both phenotypes, and also the genotypes at various SNPs in these genomic regions, have been previously reported to show a north to south gradient across Europe [4,42]. One explanation for why we did not pick-up these signals in our data might be that the genotype frequency gradients in these genomic regions are too small for detection on a micro-geographic level such as within the Netherlands using the methods we applied. In the second analysis, we performed a spatial autocorrelation analysis on individual relationships [30]. The obtained covariance matrix between individuals is indicative of a statistically significant clinal pattern across the Netherlands (Figure 6, *P* value of the autocorrelogram after 1,000 replications <0.0005). In order to infer the slope of this cline, we computed a pairwise Fst matrix between pairs of subpopulations and performed a Bearing correlogram analysis [37]. The angle at which the Mantel correlation between the pairwise Fst matrix, and the geographic-angle based distance reached its maximum was 110 degrees (r = 0.254, *P* value after 999 permutations = 0.001). This indicates a southeast to northwest orientation of the genomic cline within the Netherlands for the increase of genetic differentiation between the 54 Dutch subpopulations analyzed. A Mantel test between the geographic distance matrix and the Fst subpopulation pairwise matrix, without considering angle information, revealed a correlation of r = 0.165 (*P* value based on 999 replicates = 0.00914).

We additionally explored whether geographically restricted dialects of the Dutch language, which also show north–south gradients as reported elsewhere [43], could be associated with the genomic diversity pattern we observed across the country. We estimated the amount of genetic variation explained by classifying the 54 subpopulations according to the 6 main dialects (Frisian, Groningen, Overijssel, Southwest Limburg, Brabant and Central Dutch varieties) that were previously identified in a dendrogram analysis by Heeringa [43]. Analysis of Molecular Variance (AMOVA) showed that classifying the Dutch subpopulations by dialect explains a small and statistically nonsignificant proportion of only 0.2% (*P*(random value >observed value) = 0.99707 after 1,000 iterations) of the total genetic variance observed. This result indicates that dialects are unlikely to have influenced our genomic findings including the spatial distribution of genomic diversity across the Netherlands.

The genome-wide southeast to northwest cline in the distribution of the genomic diversity across the Netherlands observed here via different analyses could be interpreted as fitting the southeast to northwest genetic cline previously found for the whole of Europe [3,4]. Without any prior knowledge about the geological and human settlement history of the sampled region, one may explain the observed genomic gradient across the Netherlands by the major prehistoric demographic events that were previously used to explain the cline seen across the whole of Europe [1]. However, taking into account the strong palaeogeographic and archaeological evidence for marked population discontinuities on the Dutch territory during several, including more recent, periods in the Dutch history, we regard it as rather unlikely that the Palaeolithic colonization together with postglacial re-colonization and the Neolithic transformation process are directly responsible for the genomic findings we obtained here for the Dutch population. To test if the observed genomic cline could also be explained by recent events in the Dutch history [see Additional file 1 for details], we ran two SPLATCHE2 [38] simulations. In the first simulation, we used the parameters of the Palaeolithic-Neolithic model previously proposed by Francois *et al*. [5] (see Methods for details). In the second simulation, we introduced a genetic discontinuity scenario around 250 *Anno Domini* (AD) (70 generations ago, assuming 25 years per generation) in the Netherlands, when most of the country close to the sea remained uninhabitable by humans (Figure 1) up to 35 generations ago, or until approximately 1250 AD. After this period, previously uninhabitable areas acquired the same carrying capacity as the rest of Europe and became populated by individuals from the surrounding populations in this model. For each simulation, we generated 1,000 SNPs at a minimum allele frequency (MAF) of 0.03 and computed the Fst distance between pairs of populations using Arlequin 3.1 [39], setting all negative Fst values to 0. The Fst matrix of each of the two demographic models was then used in a MDS analysis and compared by means of Procrustes analysis either with the geographic coordinates or with the MDS coordinates of the other model. We found that both models strongly correlate with geography (correlation with geography in a symmetric Procrustes rotation when using the genetic discontinuity model: 0.576, *P* value = 0.001; correlation in a symmetric Procrustes rotation of the Palaeolithic-Neolithic model: 0.62, *P* value = 0.001; both analyses based on 1,000 permutations). Furthermore, we observed that the outcomes of both model simulations are statistically significant in their correlation with each other (correlation in a symmetric Procrustes rotation: 0.446, *P* value = 0.003). The Bearing correlogram analysis using the Fst distance matrix obtained with the model of genetic discontinuity is highly similar to the one produced by considering genetic continuity (Adjusted R-squared: 0.93, *P* value <2.2e-16), which suggests that the genetic gradient produced by both models is virtually indistinguishable. This finding, together with the rich archaeological evidence for human genetic discontinuity on Dutch territory led us to propose that the observed genomic gradient across the Netherlands was not caused by ancient but rather by recent events in Dutch history.

Although it cannot be excluded that the observed genomic gradient across the Netherlands that we explain by recent events, by chance resembles the ancient genomic gradient seen across Europe, another explanation is that this gradient was re-introduced by immigration of people from outside regions carrying ancient genetic signatures. One prerequisite for this scenario would be that immigration did not occur by one major population (or a limited number of populations), described as elite-dominance, but by movements of several populations from adjacent areas of similar latitudes in a way that the northern parts of the Netherlands received immigrants from northern/northeastern neighboring regions, southern parts from southern/southeastern neighboring regions, and central parts from eastern neighboring regions. Also, the mainly south–north geographic orientation of the Dutch territory provides a suitable prerequisite for such a scenario given the south–north genomic cline observed across Europe. However, there is no clear evidence provided by the archaeological records that would support such a scenario. The observation that subpopulations from the central-east of the Netherlands appeared more diverse (within and between groups) on the genome-wide level compared to all other Dutch subpopulations tested, could be indicative of recent admixture with other genetically diverse subpopulations not analyzed in our study. It would require, however, more detailed archaeological and/or historical research in addition to similarly detailed genetic information from regions outside the current Dutch political borders to disentangle the exact demographic events that shaped the current genetic variation of the Dutch population.

Besides evolutionary implications, our findings of small but detectable genomic substructure in the Dutch population, particularly the detection of geographic groups of Dutch subpopulations that can be differentiated using genome-wide data, also is of relevance for epidemiology and forensics. For future epidemiological studies, this knowledge may be relevant for (disease) gene mapping on Dutch individuals to avoid confounding effects that in principle can reveal false-positive findings. For future forensic genetic studies, the implications are two-fold. First, the detected population substructure may be considered as a correction factor when estimating match probabilities of STR profiles obtained from crime scene and suspect materials in the Netherlands. Second, our data provide evidence that in case the large number of SNPs used here can be derived from a forensic DNA sample, inferring the subregion of biogeographic ancestry within the Netherlands of an unknown may be feasible, which can provide useful investigative information to find unknown perpetrators.

## Conclusions

We have shown that despite the genetic differentiation between Dutch individuals and subpopulations sampled systematically across the country being very small, the overall genome-wide diversity tends to correlate statistically significantly with geography and that the genomic map of the Netherlands resembles the geographic map of sampling locations in all dedicated analyses we performed. Furthermore, we identified a significant southeast to northwest cline in the distribution of genomic diversity across the Netherlands, similar to earlier findings from across Europe. For the Netherlands however, the classical interpretation of the observed genetic gradient by Paleolithic-Neolithic processes is challenged by the geological, archaeological and historical evidence pointing towards population discontinuity on the Dutch territory through the ages. Our demographic simulations revealed that the expected Paleolithic-Neolithic pattern in autochthonous populations would be similar to the one produced by a recent colonization of a region from neighboring areas. Considering the evidence for population discontinuity we therefore believe that the genomic patterns we observe are caused by recent rather than ancient events in the Dutch population history. On a wider picture, our results indicate that local and more recent demographic events can produce genetic patterns strongly resembling those traditionally explained by the major prehistoric migrations. We therefore suggest that future studies pay more attention to local and more recent demographic events when explaining clinal distributions of genetic diversity. Ultimately, ancient DNA analysis of past populations in comparison with DNA analysis of contemporary populations from the same region should be used to elucidate the contribution of ancient versus recent populations to the current gene pool of the Netherlands.

## Abbreviations

AD: *Anno Domini*; BRLMM: Bayesian robust linear model with Mahalanobis distance classifier algorithm; FLDO: Forensic Laboratory for DNA Research of the Leiden University Medical Center; HWE: Hardy-Weinberg equilibrium; IBD: Identical-by-descendent; IBS: Identical-by-state; LD: Linkage disequilibrium; MAF: Minimum allele frequency; MDS: Multidimensional scaling; PCA: Principal component analysis; SNPs: Single nucleotide polymorphisms; SPA: Spatial ancestry analysis.

## Competing interests

The authors declare that they have no competing interests.

## Authors’ contributions

MK designed the study with contributions from PdK and OL, and provided resources. CB, SB, TK and PN performed experimental analyses. OL performed most statistical data analyses. MvO performed some data analyses. EA provided archaeological and historical information. PdK provided samples. OL, EA, PdK and MK wrote the manuscript. All authors read and approved the final manuscript.

## Supplementary Material

Additional file 1: Note 1, Table S1, Figure S1, S2, S3, S4 **is a document containing a supplementary note about the demographic history of The Netherlands.** It also contains Supplementary Figures 1 to 4, and a table listing the geological and cultural periods with corresponding dates and population size estimates for the Dutch area. Click here for file

Additional file 2: Table S2 listing the SNPs identified by SPA analysis with strong geographic gradients in The Netherlands. Click here for file
