# Supplementary material for: Clinal distribution of human genomic diversity across the Netherlands despite archaeological evidence for genetic discontinuities in Dutch population history
Source: Investig Genet. 2013 May 20;4:9. doi: 10.1186/2041-2223-4-9 (PMC3707805; doi:10.1186/2041-2223-4-9)

Supplementary Table 1. Schematic overview of geological and cultural periods with corresponding dates and population size estimates for the Dutch territory ([1]: [3], [2]: [2, 14], [3]: [2], [4]: [14], [5]: [11], [6]: [17]).

Geological period[1]	Cultural period			Date[2]	Population size x 1,000
Pleistocene	Paleolithic				~0[3]
Holocene	Paleolithic	Late		12800 BP - 9700 BP	~2[3]
	Mesolithic			9000 - 5300/3400 BC	
	Neolithic	Early		5300 - 4200 BC	~10[3]
		Middle		4200 - 2900 BC	
		Late		2900 - 2000 BC	
	Bronze Age	Early		2000 - 1800 BC	
		Middle		1800 - 1100 BC	
		Late		1100 - 800 BC	15-30[3]
	Iron Age	Early		800 - 500 BC	
		Middle		500 - 250 BC	
		Late		250 - 12 BC	
	Roman Period	Early		12 BC - 47 AD	
		Middle		47 - 260 AD	~150[3]
		Late		260 - 450 AD	
	Medievals	Early	Merovingian	450 - 750 AD	~40[4]
			Carolingian	750 - 950 AD	
		Late		950 -1500 AD	
	Modern period			1500 AD	~2,000[5]
				1800 AD	~2,500[5]
				1900 AD	~5,000[6]
				1950 AD	~10,000[6]
				2011 AD	~16,000[6]

Supplementary Figure 1. Estimated population size on Dutch territory over times, based on Supplementary Table 1.

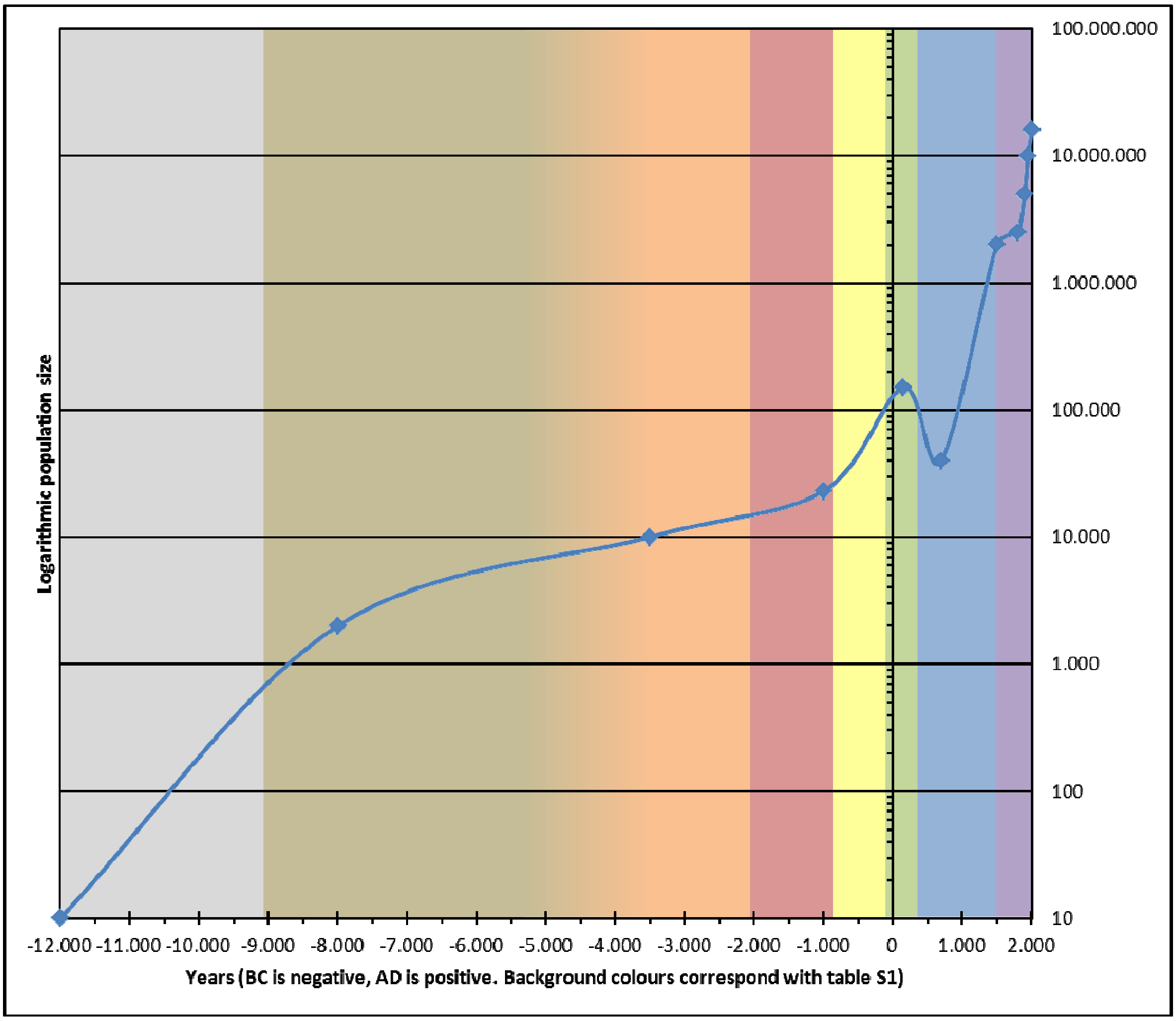

Supplementary Figure 2. A) Classical MDS on genome-wide autosomal data performed with 969 individuals of 54 Dutch subpopulations after data cleaning. B) Mclust analysis performed in the first two dimensions. Mclust detects three main clusters.

A

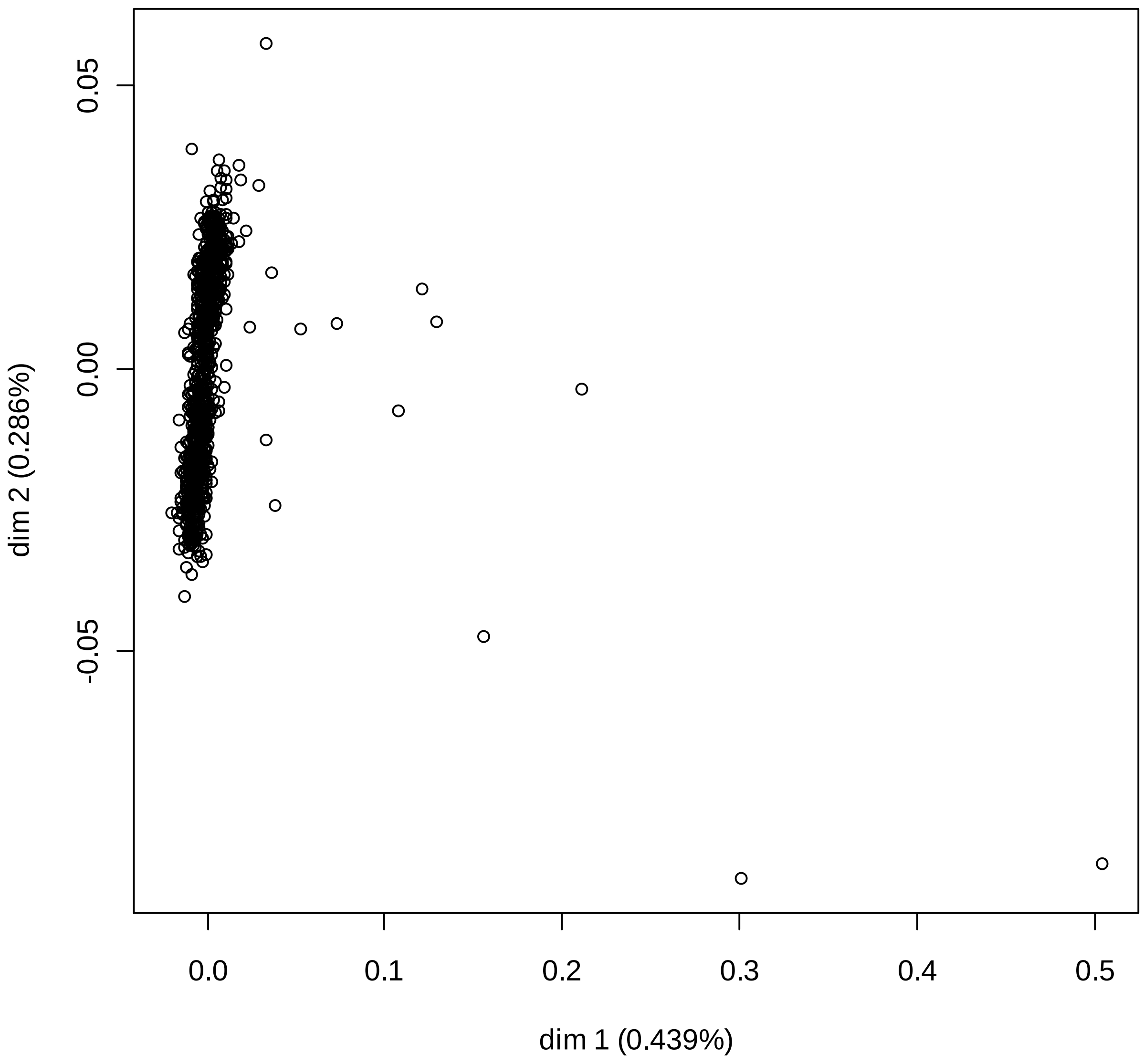

B

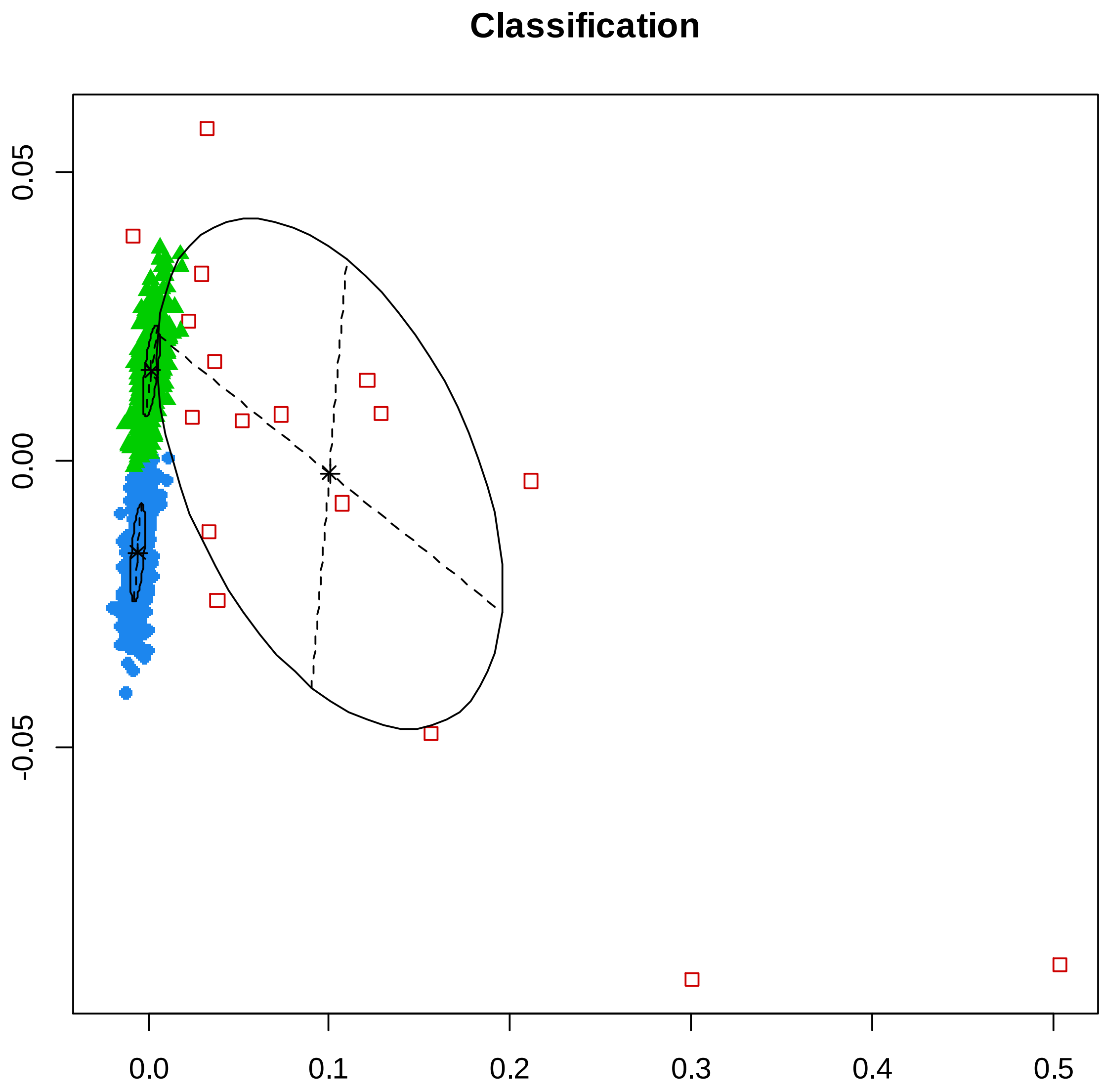

Supplement: Additional file 1: Note 1, Table S1, Figure S1, S2, S3, S4 — is a document containing a supplementary note about the demographic history of The Netherlands. It also contains Supplementary Figures 1 to 4, and a table listing the geological and cultural periods with corresponding dates and population size estimates for the Dutch area. [file 2041-2223-4-9-S1.pdf]
